# Population genetic structure of raccoons as a consequence of multiple introductions and range expansion in the Boso Peninsula, Japan

**DOI:** 10.1038/s41598-021-98029-1

**Published:** 2021-09-29

**Authors:** Miki Hirose, Kazuya Yoshida, Eiji Inoue, Masami Hasegawa

**Affiliations:** Department of Biology, Faculty of Science, Toho University, 2-2-1 Miyama, Funabashi, Chiba 274-8510 Japan

**Keywords:** Molecular biology, Population genetics, Conservation biology, Invasive species

## Abstract

The raccoon (*Procyon lotor*) is an invasive carnivore that invaded various areas of the world. Although controlling feral raccoon populations is important to reduce serious threats to local ecosystems, raccoons are not under rigid population control in Europe and Japan. We examined the D-loop and nuclear microsatellite regions to identify spatially explicit and feasible management units for effective population control and further range expansion retardation. Through the identification of five mitochondrial DNA haplotypes and three nuclear genetic groups, we identified at least three independent introductions, range expansion, and subsequent genetic admixture in the Boso Peninsula. The management unit considered that two were appropriate because two populations have already genetic exchange. Furthermore, when taking management, we think that it is important to monitor DNA at the same time as capture measures for feasible management. This makes it possible to determine whether there is a invasion that has a significant impact on population growth from out of the unit, and enables adaptive management.

## Introduction

Controlling invasive species is important for reducing threats to biodiversity and natural resources on a global scale^1^, because introduced species may disrupt local ecosystems and eliminate native species through predation and herbivory competition, pathogen transmission, or hybridization^2^. Invasion may ultimately lead to serious human health^3,4^ and economic^5,6^ problems.

Implementing biological control for invasive species often requires both systematic measures and adaptive management approaches^7^. For example, a systematic measure includes early detection and rapid response to eradicate newly established populations; monitoring population dynamics and range expansion are important components of adaptive management approaches. Not only distribution data but also molecular analyses are effective tools for understanding the process of range expansion by understanding the degree of genetic exchange under various environmental conditions^8^. Moreover, an eradication program can be adopted by identifying the marginal populations to limit range expansion and avoid genetic admixture^9^, whereas the core populations must be kept at low density to prevent negative impacts^10^. Asada^11^ proposed “lag-phase management” as an effective management technique for mammals, considering the sexual difference in dispersal distance, as males disperse over significantly longer distances than females^12^. When the target species have gender differences in dispersion distance, only males are distributed in the foremost part of the distribution expansion area, and a low-density region “lag-phase” is formed, where the Allee-effect^13^ retards population growth. Thereafter, the female spreads its distribution similar to “petals on a rose”^14^, and an “increase-phase” is formed, where the number of animals increases rapidly. Although it is difficult to achieve eradication, maintaining lag-phase by applying capture pressure is possible. We think that this lag-phase management can effectively prevent the expansion of the distribution area. For that purpose, it is important to understand the current genetic structure and set an appropriate management unit. This enables adaptive management by monitoring whether it occurs to exchange with other regions and whether countermeasures are successful.

Raccoon (*Procyon lotor*) is a carnivore originally native to North and Central America^15,16^. Due to its invasiveness in terms of wide dietary niche, habitat generalist, high density, and rapid population growth, feral raccoons are a serious threat to local ecosystems and eliminate native species through predation, competition, and pathogen transmission^2^. Despite the urgency and need for raccoon population management, it has not been properly managed in Europe^10^ and Japan^17^, because of increasing population trends, unlimited range expansion, and an inefficient management strategy^10,18^.

To take effective management, we tried to understand feasible management units for retarding population growth. We explored molecular genetic methods for understanding the process of range expansion and degree of genetic exchange in various landscapes^8^ in a geographically limited peninsular region of Japan (Boso Peninsula of Chiba Prefecture). Since the relationship between raccoon expansion process and genetic exchange in Japan might be extremely complex^19^, it is difficult to estimate the details of range expansion only from sighting records. However, if we focus on a geographically limited area, we might be able to examine the details of range expansion in association with landscape and genetic structures within a particular region.

In this study, we analyzed the mitochondrial D-loop and microsatellite loci of raccoons in the Chiba Prefecture to detect the area of introduced populations and expansion process, supplemented with information on past distribution^20,21^. We have discussed the recognition of management units for feral raccoons in a semi-confined landscape of the Boso Peninsula as a model system.

## Materials and methods

**Figure 1:**
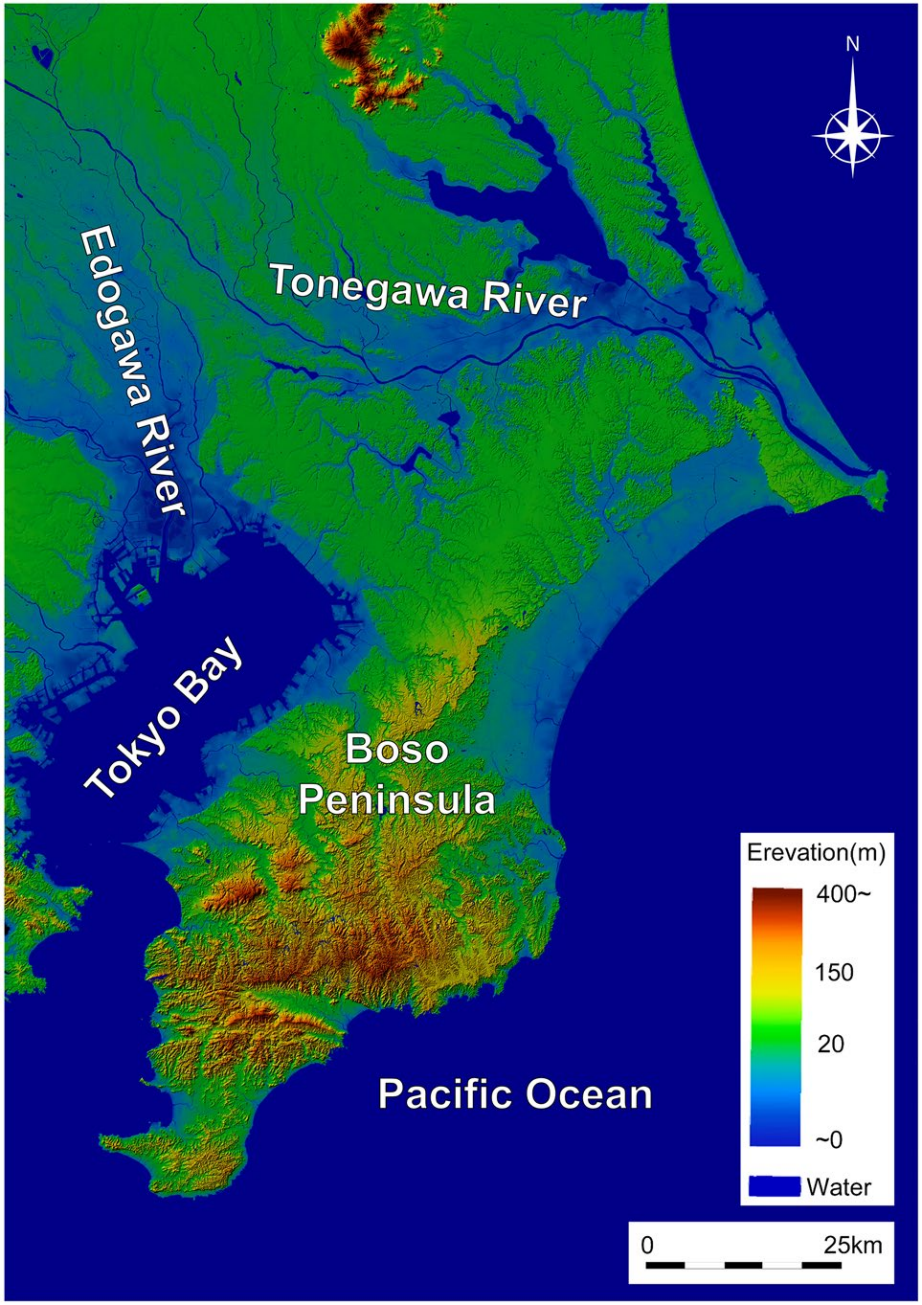
Around the study area. Partially modified based on the map of Geospatial Information Authority of Japan. The maps were created by using Microsoft Power Point office 365 based on the Digital Map published by Geospatial Information Authority of Japan website (https://maps.gsi.go.jp/)^25^.

The area of Chiba Prefecture almost coincides with the Boso Peninsula (ca. 2800 km^2^), protruding into the Pacific Ocean from the Kanto Plain (Fig. 1). The Kanto Plain, the largest plain surrounding the Tokyo metropolitan area, developed during the late Quaternary period through tectonic activities and glacio-eustatic sea-level changes^22^. The northern half of the Boso Peninsula is bordered by the Edogawa River along the northwestern margin and the Tonegawa River along the northwest to northeast margin (Fig. 1), sloping from an altitude of 100 m in the southeast to 10 m in the northwest, because the central area of the Kanto Plain is still subsiding. In contrast, the southern part of the Boso Peninsula primarily consists of ancient but still uplifting terrain with a well-dissected steep valley, although the entire terrain is usually below 300 m altitude. These hilly terrains are intervened by the narrow coastal diluvial plain and are ultimately surrounded by the Tokyo Bay and the Pacific Ocean. The northwestern and coastal bay areas of the peninsula are located within the highly developed and densely populated Tokyo metropolitan area, but the eastern and southern hilly regions are outskirts of the metropolitan area with sufficient cropland, grassland, and forest areas^23^. The climate of the peninsula is warm–temperate, with 5.2–29.0 °C mean monthly temperature and 1193–2203 mm annual precipitation, as reported in 2020^24^.

Tissue samples were collected from 179 carcasses of feral racoons that were euthanized for pest control in Chiba Prefecture from November 2014 to August 2019. Mitochondrial DNA (mtDNA) haplotype and nuclear microsatellite genotypes were determined for the tail tissue preserved in 99.5% ethanol or buccal cells collected with a cotton swab, rinsed in phosphate-buffered saline (PBS) solution (137 mM NaCl, 8.1 mM Na_2_HPO_4_, 2.68 mM KCl, and 1.47 mM KH_2_PO_4_), and preserved in medium with 70% concentration of ethanol until DNA extraction.

### DNA extraction and laboratory procedures

DNA was extracted using DNeasy Blood & Tissue Kit (QIAGEN; Tokyo, Japan). The tissue samples were cut into small pieces and DNA was extracted according to the manufacturer’s instructions. The buccal cell sample was rinsed three times in PBS solution and DNA was extracted. The DNA was eluted using 200 µL AE buffer.

A part of the mitochondrial D-loop region was amplified using PLO-L15997 (5′-CCATCAGCACCCAAAGCT-3′)^26^ and PLO-CRL1 (5′-CGCTTAAACTTATGTCCTGTAACC-3′)^27^ primers. The PCR amplifications were performed in 15 µL total volume containing 1 µl template, 0.5 U Expand High Fidelity Enzyme Mix (Roche; Tokyo, Japan), 150 µM of each dNTP, and 300 nM of each primer. After an initial incubation at 95 °C for 5 min, PCR was performed for 35–40 cycles of denaturation at 94 °C for 30 s, annealing at 56 °C for 30 s, and extension at 72 °C for 30 s, followed by a final extension at 72 °C for 30 min. The PCR products were purified using EXoSAP-IT (Affymetrix; Cleveland, OH, USA) and sequenced using a Big Dye Terminator v3.1 Cycle Sequencing Kit (Thermo Fisher Scientific; Waltham, MA, USA) using the FASMAC sequencing service (FASMAC; Kanagawa, Japan). The sequences were aligned using MEGA 10 software^28^.

A total of 24 microsatellite loci^29–31^ were analyzed via multiplex PCR (Table 1). To modify the amplicon length, new primers for three loci (PLO-M3, PLO-M17, and PLO-M15) were designed based on the sequences registered in GenBank using Primer 3^32,33^ (Table 2). We added a GTTCTT sequence to the 5′ end of the reverse primers of the loci that included dinucleotide repeats^30,31^; this sequence promotes near complete adenylation of the 3′ end and decreases genotyping error risk^34,35^. We designed three multiplex PCR sets (Multiplex 1: 7 primers, Multiplex 2: 10 primers, Multiplex 3: 7 primers) using Multiplex Manager v1.2^36^ (Table 2). We added 1 μl DNA template and 2.5 μl Multiplex PCR Master Mix (QIAGEN) to a 5 μl mixture for performing the PCR. The concentration of each primer is listed in Table 2. The amplification conditions were as follows: 15 min at 95 °C, followed by 40 cycles of 94 °C for 30 s, 57 °C for 30 s, 72 °C for 30 s, and a final extension at 60 °C for 30 min. We increased the number of cycles to 45 for the samples that did not yield adequate products after 40 PCR cycles. Fragment analysis of the PCR products was performed using the FASMAC fragment analysis service (Kanagawa, Japan) and the genotypes were determined using Peak Scanner Software v1.0 (Thermo Fisher Scientific K.K.; Tokyo, Japan).

**Table 1:** Characteristic of the microsatellite 24 locus and primer sets of multiplex PCR.

Primer set	Marker	*N*	Range	*Na*	*Ho*	*He*	Label	Concentration (μM)
Multiplex 1	PLO-M03^a^	179	122–142	7	0.536	0.545	FAM	1
	PLO-M20^a^	177	173–205	11	0.836	0.754	FAM	2
	PLO-M17^a^	179	236–256	8	0.827	0.810	FAM	2
	PLO-M02^a^	177	285–318	10	0.785	0.800	FAM	1.5
	PLO-M15^a^	179	109–143	12	0.832	0.870	HEX	1
	PLO-2-117^a^	163	297–339	14	0.840	0.850	HEX	8
	PLO2-14^a^	177	230–256	16	0.842	0.890	NED	4
Multiplex 2	PLM17^c^	178	96–110	10	0.815	0.836	FAM	2
	PLM07^c^	179	155–169	8	0.771	0.820	FAM	2
	PLOT-11^b^	178	201–217	8	0.708	0.725	FAM	2
	PLOT-08^b^	178	244–260	5	0.624	0.611	FAM	2
	PLOT-03^b^	176	302–312	6	0.699	0.740	FAM	2
	PLOT-04^b^	164	344–372	12	0.616	0.849	FAM	2
	PLM05^c^	179	98–118	7	0.626	0.667	HEX	2
	PLM14^c^	179	154–168	8	0.726	0.761	HEX	2
	PLM08^c^	178	210–222	7	0.624	0.669	HEX	4
	PLOT-05^b^	179	118–132	8	0.620	0.711	NED	2
Multiplex 3	PLM09^c^	178	105–135	11	0.758	0.795	FAM	2
	PLOT-06^b^	179	161–181	6	0.665	0.693	FAM	2
	PLOT-07^b^	174	214–222	7	0.454	0.530	FAM	2
	PLM03^c^	179	130–140	5	0.547	0.577	HEX	2
	PLOT-02^b^	179	184–212	11	0.799	0.829	HEX	2
	PLM06^c^	177	98–110	7	0.785	0.812	NED	2
	PLOT-10^b^	179	158–182	7	0.492	0.565	NED	2

*N* number of the samples, Range range of the alleles, *N* number of the alleles, *Ho* observed heterozygosity, *He* expected heterozygosity, Label Primer Dye, *Concentration (μM)* Concentration of the primer in multiplex PCR.

^a^Cullingham et al. (2006)^29^.

^b^Fike et al. (2007)^30^.

^c^Siripunkaw et al. (2008)^31^.

**Table 2:** Primer of the microsatellite region that we designed newly.

Locus	Primer sequence	Motif sequence	Accession number
PLO-M3	F-GAATGAGTCCATTTTGCTGGT	(ATCT)_15_	DQ388435
	R-CAGAACAGTGGGTGGGAGAT		
PLO-M20	F-GATTCTTATGTCTCTTGGGA	(TCTA)_17_	DQ388437
	R-AAGTGCTTCAAGAGAAAGTGC		
PLO-M17	F-CAAGGGAGAGGAAGAAGCAG	(GTTT)_3_	DQ388440
	R-CCCCTTCCCCTGTACATATTC	(TATC)_12_	

### Genotyping mtDNA

For the mtDNA control region, 406 bp sequences that overlapped with the same region used in a previous study^37^ were determined, and a median-joining network, which included the published sequences in the North American population^27^ and 15 sequences from invasive raccoons in Japan retrieved from GenBank (NC_009126, AB297804, AB361247, AB462045-49, LC455747-53), was constructed using Network 5 (http://www.fluxus-engineering.com)^38^. We followed the categorization of lineages in the network described by Cullingham et al.^27^. Thereafter, the genetic diversity (*He*) of microsatellite loci was compared among the feral raccoons from Chiba, Hokkaido^19^, those from Spain, Germany, Czech Republic, and Poland^39–42^, and the native raccoons from North America, including Ontario, Indiana, Columbia, Missouri and Illinois^29,30,41,43^ with a sign test using R version 3.6.2. Thus, we downloaded the Excel file with geographical coordinates of each animal and the allelic fragment length at every gene locus from the Dryad database (10.5061/dryad.412h3)^40^. Information regarding the genetic diversity of raccoons of Germany^40^ was calculated using GenAlEx 6.502.

### Genotyping and identification of genetic groups with microsatellite loci

Genetic diversity and differentiation among district groups of the raccoons were described initially by genotyping 179 raccoons sampled from the various localities of Chiba Prefecture, and by calculating the number of alleles (*Na*) in each locus, the effective number of alleles (*Ne*), observed heterozygosity (*Ho*), expected heterozygosity (*He*), private alleles (*pA*), and allelic richness using GenAlEx 6.502^44,45^ and FSTAT 2.9.3.2^46^. We regarded each city as a raccoon district group, and the adjacent districts of Kimitsu and Futtsu were combined due to the small sample size. Narita, Chiba, and Sosa were excluded from the analysis of the genetic differentiation due to the low sample size. The *He* among the districts were compared through a pairwise t-test using R version 3.6.2 (R Core Team 2019) and corrected via the Bonferroni method.

Individual-based genetic clustering was performed to determine the number (K) of genetically different groups (hereinafter called “cluster”) using STRUCTURE 2.3^47^. In this analysis, the admixture model and the correlated allele frequency model were used. STRUCTURE 2.3 was run with 10 repetitions of 1,000,000 iterations of MCMC simulation, following a burn-in of 200,000 iterations at K = 1–10. Using STRUCTURE Harvester^48^, we obtained K of the highest likelihood by considering the calculated ΔK. STRUCTURE software calculates the fractional membership of each animal in each cluster (Q). The mean Q was calculated from 10 trials in the animal using CLUMPP 1.1.2^49^ and the Q value of each animal was illustrated using distruct 1.1^50^. We set a criterion to include or exclude individual raccoons in particular clusters by considering the Q value of individual raccoons. We considered individual raccoons were to be from a single cluster when the highest Q value was > 0.7. Raccoons with the highest Q value of < 0.7 were grouped as a mixture that did not belong to a particular cluster. We then calculated the district groupwise cluster frequency.

## Results

### Mitochondrial D-loop

**Figure 2:**
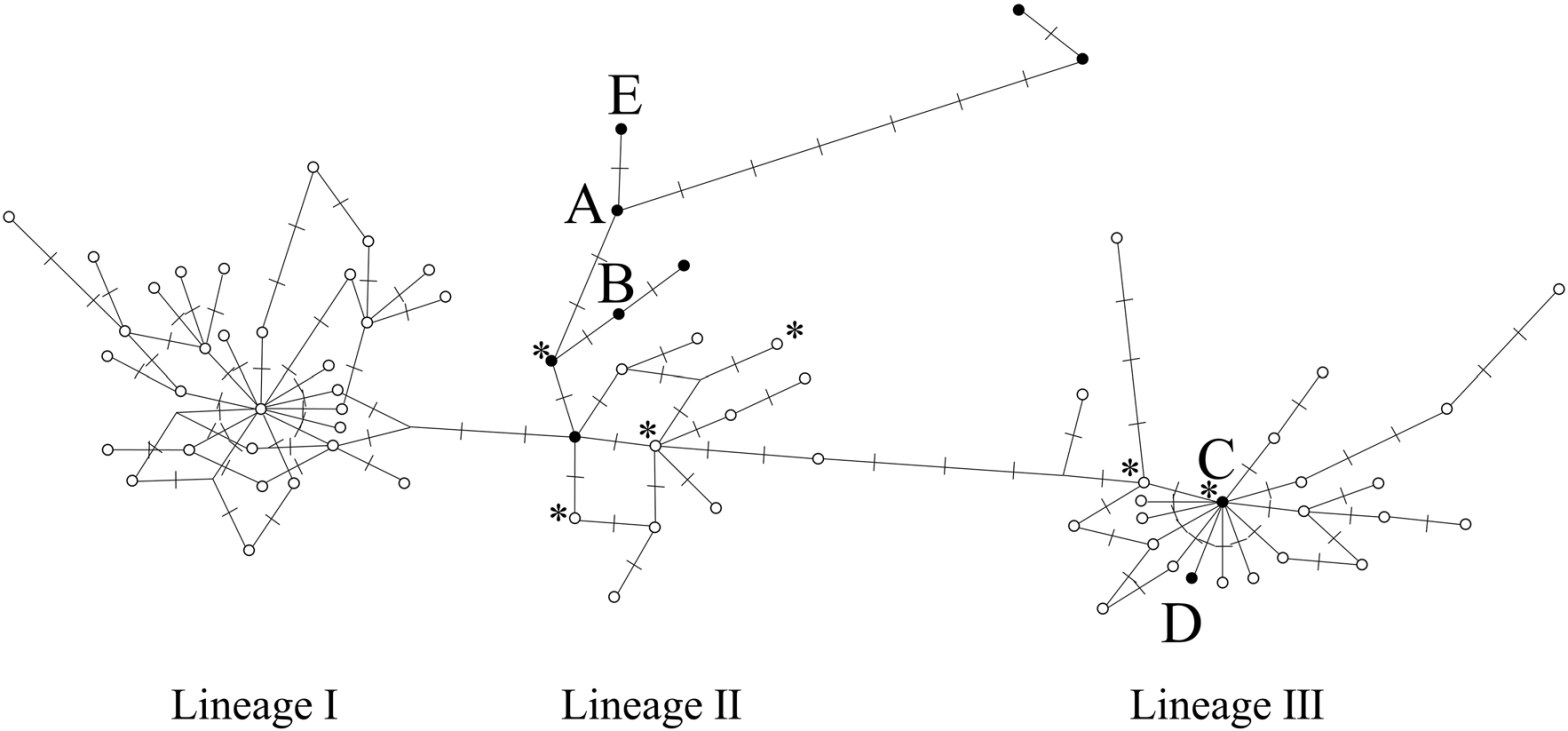
The median-joining network of mitochondrial DNA sequences of raccoons. Haplotypes of black circles (**A–E**) were detected in Chiba prefecture, Japan. White circles represent haplotypes in North American Populations by Cullingham et al.^27^ and haplotypes with an asterisk were reported in European populations^26,39,41^. We followed the categorization (lineages I, II, and III) of Cullingham et al.^27^.

**Figure 3:**
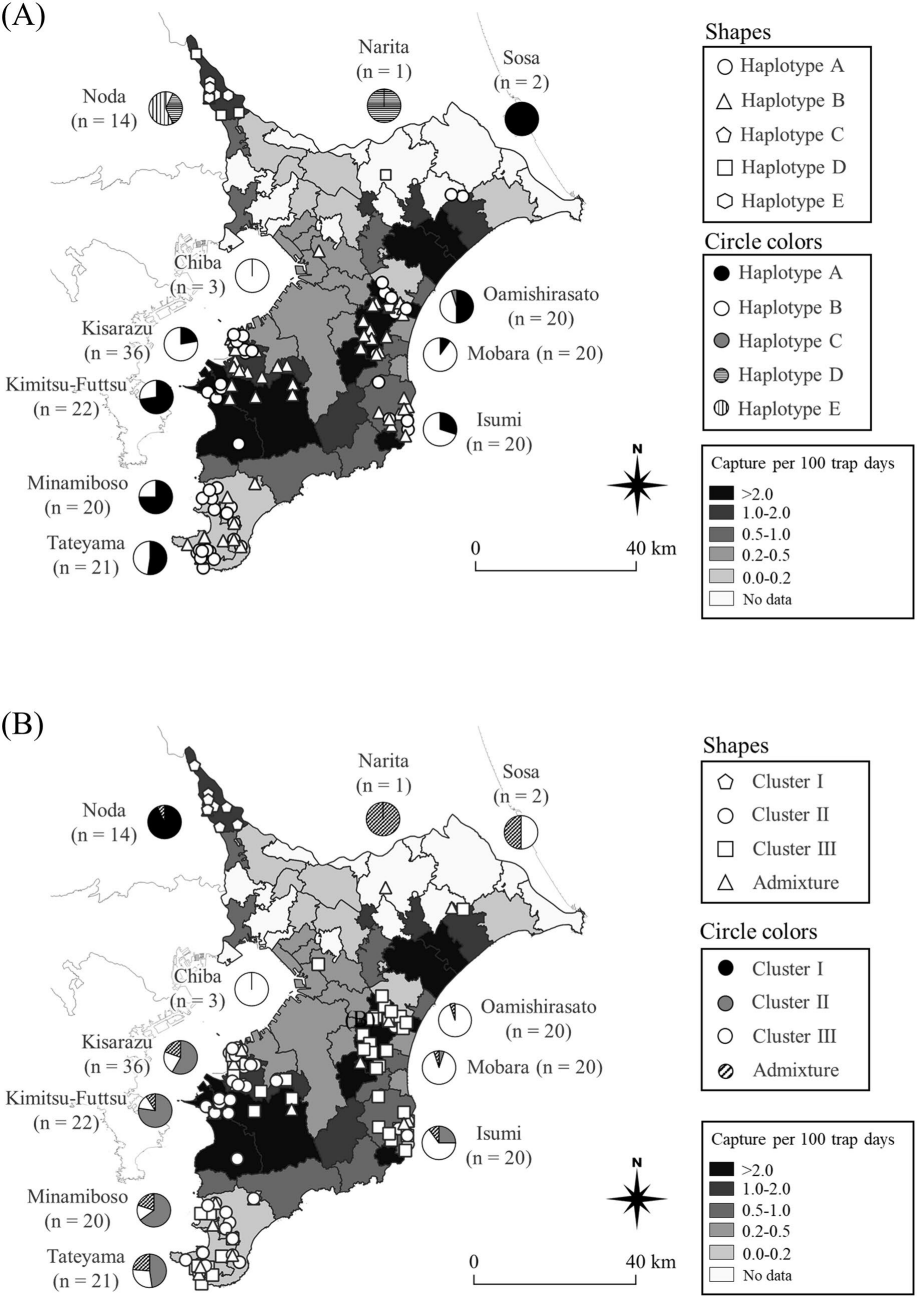
The background maps of (**A,B**) show the distribution of raccoons in Chiba prefecture, Japan in 2017. The maps are based on the CPUE (captures per 100 trap days) value in each administrative district (Chiba prefecture unpublished). (**A**) The distribution and the frequency of haplotypes observed in Chiba prefecture, Japan. (**B**) The frequency and the distribution of clusters (K = 3) in each district groups in Chiba prefecture, Japan. The maps in (**A,B**) were created by using Microsoft Power Point office 365 and QGIS ver. 3.4.4 (https://qgis.org/en/site/forusers/download.html).

Five haplotypes (A, B, C, D, and E) were identified in the Chiba Prefecture. Among them, three haplotypes (C, D, and E) were first found in Chiba and the sequences have been registered in the DNA Data Bank of Japan nucleotide sequence database (accession numbers: LC565453, LC565454, and LC565455, respectively). Only one single nucleotide polymorphism (SNP) was detected between haplotypes A and E and between haplotypes C and D. The median-joining network, including published haplotypes, is shown in Fig. 2. In Noda and Narita, located in northern Chiba, haplotypes B, D, and E were identified, with haplotypes D and E being most common. In the other district groups, haplotypes A, B, and C were identified, wherein haplotypes A and B most common (Fig. 3A).

### Microsatellite genetic diversity among district groups

Table 3 presents the genetic diversity index data for each district group. The value of *pA* was highest in Noda. The values of others were similar among district groups. No significant difference was found in *He* between district groups (Noda and Kimitsu-Futtsu, *P* = 0.86; Noda and Minamiboso, *P* = 0.87; all the other dyads, *P* = 1).

**Table 3:** Genetic diversity of each district for raccoons in Chiba prefecture, Japan.

District	*N*	*Na*	*Ne*	*AR*	*Ho*	*He*	*pA*
Noda	13.58	6.58	4.34	5.18	0.74	0.75	22
Oamishirasato	19.96	6.33	3.91	4.54	0.70	0.69	4
Mobara	19.75	6.46	3.97	4.66	0.72	0.70	3
Isumi	19.88	5.88	3.84	4.44	0.70	0.70	2
Kisarazu	35.79	6.25	3.96	4.42	0.69	0.71	4
Kimitsu-Futtsu	21.67	5.50	3.46	4.17	0.69	0.67	1
Minamiboso	19.71	5.75	3.55	4.30	0.67	0.67	0
Tateyama	20.54	5.75	3.81	4.44	0.72	0.71	0

*N* mean of the number, *Na* estimated number of the alleles, *Ne* effective number of alleles, *AR* mean of the allelic richness, *Ho* observed heterozygosity, *He* expected heterozygosity, *pA* number of the private alleles.

### Comparison of the microsatellite genetic diversity with other areas

Table 4 shows a comparison of the mean *He* in Chiba Prefecture and that reported in previous studies. The number of loci used for comparison was different because the markers used in different studies were different. There is no significant difference between the mean *He* between Chiba Prefecture, native areas, central Europe, Germany, and Hokkaido. Although the mean *He* in Spain was not significantly different from that in the Chiba Prefecture, the genetic diversity tended to be lower than that in the Chiba Prefecture (*P* = 0.07).

**Table 4:** The comparison results of the genetic diversity for raccoons between Chiba prefecture, Japan and other areas.

	Area	Number of loci	*He*	*He* of Chiba	*P-*value	Theses
Native area	Ontario	7	0.84	0.79	1.00	Cullingham et al. (2006)^29^
	Indiana	9	0.80	0.70	0.18	Fike et al. (2007)^30^
	Illinois	7	0.83	0.79	1.00	Santonastaso et al. (2012)^43^
	Missouri	8	0.81	0.74	0.29	Alda et al. (2013)^41^
Invasion area	Germany, Czech, and Poland	11	0.66	0.71	0.23	Biedryzycka et al. (2014)^42^
	Germany	16	0.70	0.72	0.21	Fischer et al. (2015)^40^
	Spain	8	0.62	0.74	0.07	Alda et al. (2013)^41^
	Hokkaido	5	0.78	0.76	1.00	Okuyama et al. (2020)^19^

*Number of loci* the number of loci that we used for comparison, *He* mean of expected heterozygosity, *P-*value The P-value that was calculated a result of sign test.

### Microsatellite genetic structure

**Figure 4:**
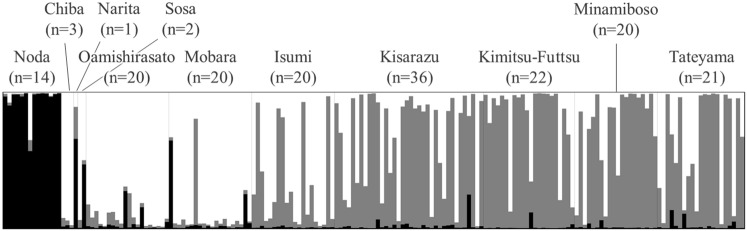
The fraction of the cluster was estimated by STRUCTURE (K = 3). The individuals are expressed in one bar. The color of cluster I is black, cluster II is grey and cluster III is white. The length of color bars expresses a fraction of the cluster (*Q* value).

Based on the ΔK value, the most probable structure clustering was K = 3. Figure 3B shows the admixture frequency and frequency of animals with Q value > 0.7. Three clusters were estimated for the entire Chiba Prefecture. The Noda district group comprised Cluster I and a few admixture animals. The other district group comprised clusters II, III, and an admixture. Clusters II and III dominated the southeast and southwest areas, respectively. Figure 4 shows the fraction of the cluster (Q value) of each animal. Only one admixture with high Cluster I proportion was detected in Noda, Narita, Sosa, and Mobara district groups. The other admixtures included clusters II and III. Table 5 shows the correspondence between the clusters and mitochondrial haplotypes. Most animals in Clusters I, II, and III had haplotypes E, A, and B, respectively.

**Table 5:** The correspondence of clusters and haplotypes for raccoons in Chiba prefecture, Japan.

Cluster	Haplotype					Total
	A	B	C	D	E	
I	0	1	0	4	8	13
II	36	31	0	0	0	67
III	25	49	0	0	0	74
Admixture	9	13	1	2	9	25
Total	70	94	1	6	8	179

The numerals are the number of individuals.

## Discussion

The feral raccoon, both in Japan and Europe, originated initially from animals imported from North America through pet trade^10,17,19^. The Invasive Alien Species Act of Japan enacted in 2005 has banned the pet trade of raccoon, and strictly regulated the transportation of living animals within Japan since 2005. Thus, the newly released or escaped captive raccoons or animals dispersed from adjacent areas should account for the origin of raccoon populations in Boso Peninsula. Boso Peninsula is almost isolated from the adjacent prefecture by the sea and the large rivers of Tonegawa River and Edogawa River (Fig. 1); thus, either human-induced re-introduction from other areas or released or escaped captive pets should be the primary sources of feral raccoons in Boso Peninsula. Based on this unique geographic situation, we have discussed the number of possible introductions and subsequent genetic admixture processes among the locations of multiple releases or escapes by referencing the spatial genetic structure revealed in this study.

Based on the mtDNA haplotype analysis and historical records of feral raccoon, Yoshida et al.^37^ hypothesized that two independent expansions have occurred from the different founder populations, with a second expansion after the first founder had already spread over the Boso Peninsula. An alternative possibility is that, after the single release of several raccoons with different mtDNA haplotypes, genetic drift during the expansion process generated local genetic differentiation. Both processes result in spatial genetic structure after the initial expansion; however, their outcomes are different. If multiple releases with low genetic diversity within the founder animals occur at different locations, genetic admixture among the multiple founders during the subsequent expansion would eventually diminish regional genetic differentiation. Alternatively, if a single release event with high genetic diversity within the founder animals occurs in a single location, the genetic differentiation observed during the expansion process is preserved.

We detected five mtDNA haplotypes within the Chiba Prefecture, two of which have already been reported by Yoshida et al.^37^ in southern Boso Peninsula. The three newly detected haplotypes were present in tissue samples collected from the raccoons of northern Boso Peninsula. However, the SNPs between haplotypes A and E and haplotypes C and D possibly appeared after the invasion because only one SNP was identified among in each haplotype pair. This means that a third invasion event occurred in addition to the first and second invasion events in the southern Boso Peninsula, suggesting that at least three mitochondrial haplotypes were introduced to the Chiba Prefecture or expanded from outside.

Three genetic groups (clusters I, II, and III) were detected in the nuclear DNA of raccoons of the Chiba Prefecture using STRUCTURE analysis, and these three nuclear genetic groups corresponded to mtDNA haplotypes E, A, and B, respectively. Therefore, these three groups, two in the east (Isumi, Cluster III and haplotype B) and west (Kimitsu-Futtsu, Cluster II and haplotype A) of southern Boso Peninsula, and one in the northwest corner (Noda, Cluster I and haplotype E) of the Chiba Prefecture (Fig. 3), are compatible with the hypothesis of at least three independent introductions within the Chiba Prefecture. Historical records of raccoon distribution and abundance in the Chiba Prefecture^20,21,37^ also support the hypothesis of three independent sequential introductions and subsequent range expansion, population increase, and genetic admixture. According to Ochiai et al.^20^, colonization of raccoons was confirmed until 2001 only around Isumi. However, according to Asada^21^, raccoons inhabited at a high density not only around Isumi but also around Kimitsu-Futtsu, and Noda in 2012. In Tateyama and Minamiboso, raccoons were rarely inhabited until 2012, but now they are inhabited in large numbers. The populations of Tateyama and Minamiboso have no genetic difference from Kimitsu-Futtsu and are thought to have expanded their distribution and invaded^37^. Therefore, in Chiba Prefecture, the feral raccoons were first confirmed around Isumi in the 1990s. The next invasion events happened around Kimitsu-Futtsu and Noda from 2002 to 2012.

Even though there are significant gaps in the abundance and genetic compositions among the northwestern and southern groups (Fig. 3), the geographic structure of distribution, abundance, and genetic composition detected in this study supports the idea that these populations are exchanging extensively in Chiba Prefecture. The raccoons initially introduced to Isumi and Kimitsu-Futtsu were capable of steadily expanding their distribution in every direction, and genetic exchange occurred in the Tateyama-Minamiboso area, as already mentioned; finally, the genetic structure will disappear. We expect that the currently recognizable genetic clusters will not persist soon due to the absence of a genetic barrier between southern and northern Chiba Prefecture.

Due to the male-biased long-distance dispersal in raccoons^12,51^, genetic admixture extent differs between the sexes. Variation in mtDNA haplotypes is transmitted maternally; hence, an admixture of mtDNA haplotypes would be much slower than the nuclear genetic variations. In the case of the genetic admixture of mtDNA haplotypes between Isumi and Kimitsu-Futtsu in the Tateyama-Minamiboso area, the straight-line distance from Isumi to Tateyama is ca. 50 km, and the female range expansion proceeds with an average speed of ca. 2.9 km per year from 1998 to 2015, being much faster than the estimated speed of 0.6 km in the native area^12^.

To exterminate and manage alien species, it is necessary to set up a management unit for the target population in consideration of the movement of organisms to prevent re-invasion^52^. By analysis of nuclear DNA, it was shown that the raccoon population in Chiba Prefecture is currently divided into a northern population, a southwestern population, and a southeastern population. A wide range of gene flow has already occurred in the southern part of Chiba Prefecture. On the other hand, there is no genetic exchange yet between the northern and southern parts. Accordingly, in Chiba Prefecture, we think that it is possible to further suppress the rate of distribution expansion by setting the northern population and the southern population as separate management units and focusing on capture measures from the breaks in the distribution of each population.

When taking management, we think that is important to monitor DNA at the same time as capture measures. To reduce the distribution area of the target population and reduce the density, there must be no invasion from the outside, especially female invasion that has a huge influence on the increase in the population. Haplotypes of mtDNA between the northern populations and the southern population in Chiba Prefecture are not mixed. Therefore, by monitoring mtDNA derived from females, it is possible to determine whether or not females have invaded from another population.

Because the expansion speed of the feral raccoons is very fast, it is essential to adjust the measures in large administrative units such as Chiba Prefecture, instead of the measures in narrow administrative units where the methods and efforts differed for each municipality as current. In this study, we showed Chiba Prefecture for the model area, we believe that these ideas are not limited to raccoons but can be applied to population management measures for many alien species.
